# Risk factors and molecular epidemiology of fecal carriage of carbapenem resistant Enterobacteriaceae in patients with liver disease

**DOI:** 10.1186/s12941-023-00560-8

**Published:** 2023-01-29

**Authors:** Fangbing Tian, Yin Li, Yue Wang, Bing Yu, Jianxin Song, Qin Ning, Cui Jian, Ming Ni

**Affiliations:** 1 Department of Infectious Diseases, Tongji Hospital, Tongji Medical College, Huazhong University of Science and Technology, Wuhan, China; 2 Department of Laboratory Medicine, Tongji Hospital, Tongji Medical College, Huazhong University of Science and Technology, Wuhan, China; 3 Department of Pathogen Biology, School of Basic Medicine, Tongji Medical College, Huazhong University of Science and Technology, Wuhan, China

**Keywords:** Carbapenem resistant *Enterobacteriaceae*, Fecal carriage, Risk factor, Carbapenemase, Colonization

## Abstract

**Background:**

Carbapenem resistant Enterobacteriaceae (CRE) colonization is a risk factor for CRE infection. CRE infection results in an increase in mortality in patients with cirrhosis. However, minimal data regarding the prevalence and the risk factors of CRE colonization in patients with liver disease yet without liver transplantation are available. The present study aimed to investigate the prevalence, risk factors and molecular epidemiology characteristics of CRE fecal carriage among patients with liver disease.

**Methods:**

Stool specimens from 574 adult inpatients with liver disease were collected from December 2020 to April 2021. CRE were screened using selective chromogenic agar medium and identified by the Matrix-Assisted Laser Desorption/Ionization Time-of-Flight Mass Spectrometry (MALDI-TOF MS). Antimicrobial susceptibility was determined using the broth microdilution method. Carbapenemase genes were characterized by polymerase chain reaction (PCR) and DNA sequencing. Multilocus sequence typing (MLST) was performed for Carbapenem Resistant *Klebsiella pneumoniae* (CR-KPN) isolates and Carbapenem Resistant *Escherichia Coli* (CR-ECO) isolates.

**Results:**

The total number of stool specimens (732) were collected from 574 patients with liver disease. 43 non-duplicated CRE strains were isolated from 39 patients with a carriage rate of 6.79% (39/574). The carriage rate was 15.60% (17/109) in patients with acute-on-chronic liver failure (ACLF). Multivariate analysis indicated that ACLF (*P* = 0.018), the history of pulmonary infection within past 3 months (*P* = 0.001) and the use of third generation cephalosporin/β-lactamases inhibitor within past 3 months (*P* = 0.000) were independent risk factors of CRE colonization in patients with liver disease. *Klebsiella Pnuemoniae* (KPN) (51.28%) and *Escherichia coli* (ECO) (30.77%) were main strains in these patients. All CRE strains showed high resistance to most antimicrobials except for polymyxin B and tigecycline. Most (83.72%, 36/43) of the CRE carried carbapenemase genes. *bla*_*KPC-2*_ was the major carbapenemase gene. The molecular epidemiology of KPN were dominated by ST11, while the STs of ECO were scattered.

**Conclusions:**

The present study revealed that CRE fecal carriage rates were higher in patients with ACLF than in patients without liver failure. ACLF, the history of pulmonary infection within past 3 months and the use of third generation cephalosporin/β-lactamases inhibitor within past 3 months were independent risk factors of CRE colonization in patients with liver disease. Regular CRE screening for hospitalized patients with liver disease should be conducted to limit the spread of CRE strain.

**Supplementary Information:**

The online version contains supplementary material available at 10.1186/s12941-023-00560-8.

## Background

The negative impact of bacterial infections in liver disease is clinically relevant in any stage of the disease process [1–3]. A multicenter prospective intercontinental study showed that infection by Gram negative bacterial, such as *Escherichia coli* (ECO) and *Klebsiella pneumoniae* (KPN), is the highest in Asia patients with cirrhosis and the prevalence of multidrug-resistant (MDR) bacteria was 50% [4]. Carbapenem is the antimicrobials of last resort that are recommended for the empirical treatment for *Enterobacteriaceae* infection in end-stage liver disease (ESLD) [5]. However, since the first report in the United States in 1996 that KPN could achieve carbapenem resistance through the production of carbapenemase, carbapenem resistant *Enterobacteriaceae* (CRE) has emerged as a public health problem and is classified as an urgent threat [6]. Because of the increasing prevalence and limitation of treatment options, CRE is considered by clinicians to be highly problematic. According to the CHINET surveillance of bacterial resistance data, the rates of carbapenem resistance in ECO and KPN in China increased from 0 and 2.9% in 2005 to 2% and 24.4% in 2021, respectively [7]. It was reported that the rate of CRE isolated from patients with cirrhosis was 20% in Asia, significantly higher than the global level (9%) [4]. CRE infection resulted in increased mortality in patients with cirrhosis due to limited therapeutic options [4].

CRE colonization was found to be significantly associated with subsequent CRE infection [8–10]. In liver transplant recipients, CRE colonized patients were found to be significantly more likely to develop subsequent infection compared to noncolonized patients [11]. Implementation of enhanced infection control measures (active surveillance, contact precaution, environmental cleaning, education and hand hygiene) reduced colonization rates by carbapenem-resistant bacteria in solid organ transplantation recipients [12]. However, as a potential recipient population for liver transplantation, minimal data regarding the prevalence and the risk factors of CRE colonization in patients with various liver diseases are available. It is also unclear whether infection control measures need to be enhanced in this population. Therefore, the prevalence and possible risk factors associated with gastrointestinal colonization of CRE were evaluated in hospitalized patients as above in the largest university hospital of Wuhan, China. Additionally, a surveillance of molecular epidemiology of CRE strains colonized in stool samples were conducted in the present study.

## Methods

### Aim, research setting and design

This study was carried out at Tongji Hospital, the largest teaching hospital in central China. 574 adult patients (≥ 18 years old) with liver disease who had been admitted to the department of infectious diseases from December 2020 to April 2021 were recruited with the aim of investigate risk factors and molecular epidemiology characteristics of CRE fecal carriage among patients with liver disease yet without liver transplantation. Patients, who had been infected with CRE, discharged within 24 h or without complete information, were excluded. Fecal samples (once a week) of the recruited patients were collected and CRE were screened.

### Specimen collection and identification

Fecal samples were obtained from recruited patients and screened for CRE with selective chromogenic agar medium (Zhengzhou Dianshi biotechnology Co., Ltd., China). Cultured isolates were identified by the Matrix-Assisted Laser Desorption/Ionization Time-of-Flight Mass Spectrometry (MALDI-TOF MS, Bruker Daltonics Inc., Billerica, Massachusetts), and then carbapenem antimicrobial susceptibility testing was performed to confirm CRE using the broth microdilution method. *Enterobacteriaceae* that were resistant to meropenem or imipenem were classified as CRE.

### Antimicrobial susceptibility testing

According to the Clinical and Laboratory Standards Institute (CLSI) guidelines, antimicrobial susceptibility test was performed using the broth dilution method to determine MICs of cefotaxime, ceftazidime, cefepime, cefuroxime, cefazolin, piperacillin, piperacillin/tazobactam, amoxicillin-clavulanic acid, cefoperazone/sulbactam, ceftazidime/avibactam, imipenem, meropenem, ertapenem, levofloxacin, ciprofloxacin, gentamicin, amikacin, doxycycline, co-trimoxazole, fosfomycin, aztreonam, tigecycline, and polymyxin B. All antimicrobials, except tigecycline and polymyxin B, were interpreted according to the standard of the CLSI document [13]. For tigecycline and polymyxin B, the European Committee on Antimicrobial Susceptibility Testing (EUCAST) breakpoint was used [14]. *Escherichia coli* ATCC 25,922 and *Klebsiella pneumoniae* ATCC700603 were used as quality control standards.

### Screening for carbapenemase and other resistance genes

For CRE strains, polymerase chain reaction (PCR) was used to detect carbapenem resistance genes (*bla*_*KPC*_*, bla*_*NDM*_*, bla*_*OXA-48*_*, bla*_*VIM*_* and bla*_*IMP*_) and polymyxin resistance gene (*mcr-1*). The PCR products were sequenced and analysed using BLAST (https://blast.ncbi.nlm.nih.gov/Blast.cgi).

### Multilocus sequence typing (MLST)

MLST was performed for the genetic relationship according to the previous protocol described. Seven pairs of housekeeping genes of KPN (*rpoB, gapA, mdh, pgi, phoE, infB, tonB*) and ECO (*adk, fumC, icd, purA, gyrB, recA, mdh*) were amplified and sequenced respectively. Alleles and sequence types (STs) were assigned based on the MLST database (https://bigsdb.pasteur.fr/cgibin/bigsdb/bigsdb.pl?db=pubmlst_klebsiella_seqdef&page=profiles; https://pubmlst.org/bigsdb?db=pubmlst_escherichia_seqdef&page=sequenceQuery).

### Risk factors for CRE colonization

The case data of the colonization group and the non-colonization group were collected and included demographic information, whether liver failure and/or complications occurred, underlying/comorbid diseases, history of hospitalization, history of invasive procedures, history of infection, history of steroids use, immunization inhibitor use and antibiotic use within the past 3 months in addition to the main laboratory test indicators [liver function, blood routine examination, coagulation function, C-reactive protein (CRP) and procalcitonin (PCT)] during hospitalization.

### Statistical analysis

Categorical variables were presented as number (%) and compared with chi-square test or Fisher's exact test; Quantitative data were tested for normality firstly, and the normal distribution variables were described using mean ± standard deviation ($\bar{x}$ ± SD) after which a correlation analysis was performed using a T-test, otherwise, the non-normal distribution variables were described using median (M) and interquartile Range (IQR) after which a then correlation analysis was performed using a rank sum test. Univariate analysis was used to assess the relevant risk factors of CRE colonization, and multivariate regression analysis were then performed on variables with a value of *P* < 0.05. All *P*-values were two-tailed, and *P* < 0.05 was considered statistically significant. All analyses were performed with SPSS 26.0 (IBM Corp., Armonk, NY, USA).

## Results

### Distribution and risk factors of CRE colonization

In the present study, a total of 732 fecal samples from 574 patients were collected. 43 non-duplicate samples from 39 patients were detected as CRE positive with a carriage rate of 6.79% (39/574). The 43 CRE strains included *Klebsiella pneumoniae* (n = 20, 46.51%), *Escherichia coli* (n = 12, 27.91%), *Klebsiella oxytoca* (n = 2, 4.65%), *Enterobacter cloacae* (n = 1, 2.33%), *Enterobacter aerogenes* (n = 1, 2.33%), *Citrobacter brucei* (n = 1, 2.33%), *Citrobacter fraudii* (n = 1, 2.33%), *Raoultella planticola* (n = 1, 2.33%), *Raoultella ornithinolytica* (n = 4, 9.30%).

By querying the medical records of each patient, complete data of 547 patients were finally obtained (39 in the colonization group and 508 in the non-colonization group). Demographic information, underlying/comorbid diseases, history of hospitalization, infection and invasive procedures within the past 3 months, use of steroids, immunosuppressants, and/or antimicrobials within the past 3 months, whether acute-on-chronic liver failure (ACLF) [15] and its complications occurred in addition to the main laboratory test indicators (liver function, blood routine, coagulation function, CRP and PCT) during hospitalization were compared between the colonization group and non-colonization group, as shown in Table 1. The CRE fecal carriage rate was 15.60% (17/109) in patients with ACLF. The univariate analysis indicated that the history of hospitalization (*P* = 0.049), pulmonary infection (*P* = 0.000) and use of third generation cephalosporin/β-lactamases inhibitor (*P* = 0.000) within the past 3 months, development of ACLF (*P* = 0.000) and combination with spontaneous peritonitis (*P* = 0.000) were significantly more frequent in the CRE colonization group. The hospitalization time of the colonization group was longer than in the non-colonization group (*P* = 0.000). Further multivariate logistic regression analysis revealed that ACLF (*P* = 0.018), the history of pulmonary infection within past 3 months (*P* = 0.001) and the use of third generation cephalosporin/β-lactamases inhibitor within past 3 months (*P* = 0.000) were independent risk factors of CRE colonization in patients with liver disease (Table 2). Among 39 patients with CRE colonization, 26 patients were detected at admission (9 ACLF patients, 17 non-ACLF patients), 13 patients were detected during hospitalization (8 ACLF patients, 5 non-ACLF patients), and the details of these 13 patients were shown in Additional file 1: Table S1. Comparing the relevant laboratory indicators between the colonization group and the non-colonization group, it demonstrated that the colonization group had poorer liver function, poorer coagulation function, and higher hemogram and inflammatory indices, and the difference was statistically significant (*P* < 0.05) (Table 3).

**Table 1:** Univariate analysis of risk factors for CRE colonization in patients with liver disease

Variable	CRE colonization (N = 39)	CRE Non-colonization (N = 508)	*P-*value
Patient characteristics			
Man gender	29 (74.36%)	368 (72.44%)	0.796
Age, median(IQR)	53.77 ± 2.07	53 (46–63)	0.675
Duration of Hospitalization(days)	30 (17–43)	11 (7–19)	0.000
Hospitalization in prior 3 months			
General wards admission	30 (76.92%)	310 (61.02%)	0.049
ICU admission	0 (0.00%)	2 (0.39%)	1.000
Liver diseases			
ACLF	17 (43.59%)	92 (18.11%)	0.000
Spontaneous peritonitis	16 (41.03%)	91 (17.91%)	0.000
Hepatic encephalopathy	5 (12.82%)	39 (7.68%)	0.405
Hepatorenal syndrome	2 (5.13%)	28 (5.51%)	1.000
Underlying diseases			
Hypertension	10 (25.64%)	102 (20.08%)	0.407
Diabetes	2 (5.13%)	73 (14.37%)	0.106
Cardiovascular disease	0 (0.00%)	14 (2.76%)	0.600
Cerebral infarction	2 (5.13%)	12 (2.36%)	0.597
COPD	0 (0.00%)	6 (1.18%)	1.000
Chronic kidney disease	0 (0.00%)	3 (0.59%)	1.000
Malignant tumors	3 (7.69%)	24 (4.72%)	0.659
Hematological tumor	0 (0.00%)	8 (1.57%)	1.000
Tuberculosis	1(2.56%)	14 (2.76%)	1.000
AIDS	0 (0.00%)	0 (0.00%)	–
Invasive procedure in prior 3 months			
Surgery	3 (7.69%)	68 (13.39%)	0.308
Deep venous catheterization	0 (0.00%)	4 (0.79%)	1.000
Tracheal intubation	0 (0.00%)	1 (0.20%)	1.000
Tracheotomy	0 (0.00%)	0 (0.00%)	–
Thoracentesis/Catheterization	1 (2.56%)	6 (1.18%)	0.406
Paracentesis/Catheterization	2 (5.13%)	22 (4.33%)	1.000
Bone marrow aspiration/Biopsy	0 (0.00%)	2 (0.39%)	1.000
Lumbar puncture	0 (0.00%)	0 (0.00%)	–
Liver aspiration/Biopsy	0 (0.00%)	6 (1.18%)	1.000
Urinary catheter	0 (0.00%)	2 (0.39%)	1.000
Bronchoscopy	0 (0.00%)	0 (0.00%)	–
Gastroscopy	0 (0.00%)	7 (1.38%)	1.000
Colonoscopy	0 (0.00%)	3 (0.59%)	1.000
Blood Dialysis	0 (0.00%)	0 (0.00%)	–
Artificial liver support	2 (5.13%)	18 (3.54%)	0.948
Organ Transplant	0 (0.00%)	0 (0.00%)	–
Infections in prior 3 months			
Pulmonary infection	12 (30.77%)	49 (9.65%)	0.000
Urinary tract infection	2 (5.13%)	35 (6.89%)	0.927
Other infections	3 (7.69%)	23 (4.53%)	0.614
Use of immunosuppressants	4 (10.26%)	34 (6.69%)	0.605
Use of steroids	2 (5.13%)	17 (3.35%)	0.895
Antibiotic use in prior 3 months			
Semi-synthetic penicillins/β-lactamase inhibitor	0 (0.00%)	20 (3.94%)	0.412
Cephalosporins	0 (0.00%)	15 (2.95%)	0.562
Third generation cephalosporins/β-lactamase inhibitor	12 (30.77%)	27 (5.31%)	0.000
Carbapenem	0 (0.00%)	14 (2.74%)	0.600
Fluoroquinolone	4 (10.26%)	22 (4.33%)	0.199
Aminoglycosides	0 (0.00%)	2 (0.39%)	1.000
Tigecycline	0 (0.00%)	5 (0.98%)	1.000
Teicoplanin	0 (0.00%)	7 (1.38%)	1.000
Linezolid	0 (0.00%)	3 (0.59%)	1.000
Polymyxin	0 (0.00%)	0 (0.00%)	–
Vancomycin	0 (0.00%)	0 (0.00%)	–
Antianaerobic	0 (0.00%)	2 (0.39%)	1.000
Sulfonamides	0 (0.00%)	0 (0.00%)	–
Anti-fungal regimen	0 (0.00%)	5 (0.98%)	1.000
Macrolides	0 (0.00%)	0 (0.00%)	–
Combined use of antimicrobials (≥ 2 antimicrobials)	3 (7.69%)	24 (4.72%)	0.659

*COPD* chronic obstructive pulmonary disease, *AIDS* acquired immunodeficiency syndrome

**Table 2:** Multivariate logistic analysis of risk factors for CRE colonization in patients with liver disease

Variables	*P- value*
ICU admission in prior 3 months	0.999
Invasive procedure in prior 3 months	0.143
Long-term use of immunosuppressants	0.891
Long-term use of steroids	0.877
General wards admission in prior 3 months	0.939
ACLF	0.018
Spontaneous peritonitis	0.072
Pulmonary infection in prior 3 months	0.001
Third generatio cephalosporins/β-lactamase inhibitor use in prior 3 months	0.000

**Table 3:** Comparison of laboratory indicators between the colonization group and the non-colonization group

Variable (Referrence)	CRE colonization (N = 39)	CRE Non-colonization (N = 508)	*P-*value
Liver function			
ALB (35.0–52.0 g/L)	30.9 ± 0.8	33.6 (30.2–38.2)	0.000
ALT (≤ 41 U/L)	51 (27–105)	45 (23–146)	0.784
AST (≤ 40 U/L)	73 (44–116)	62 (35–136)	0.468
TBIL (≤ 26.0 umol/L)	124.5 (54.7–253.7)	42.2 (15.7–163.9)	0.003
DBIL (≤ 8.0 umol/L)	108.3 (30.2–201.1)	26.6 (7.8–139.0)	0.002
IBIL (≤ 16.8 umol/L)	20.1 (10.3–47.4)	12.2 (6.4–26.8)	0.011
Coagulation Function			
INR (0.80–1.20)	1.36 (1.15–1.80)	1.20 (1.05–1.51)	0.023
PTA (75.0%–125.0%)	63.9 ± 3.9	75.5 (54.0–92.0)	0.029
Blood routine examination			
WBC (3.50–9.50 × 10^9^/L)	6.22 (5.04–9.99)	5.15 (3.50–7.41)	0.006
N (1.80–6.30 × 10^9^ /L)	3.91 (3.02–8.54)	3.05 (1.94–5.13)	0.002
N% (40.0%–75.0%)	71.3 ± 2.2	64.7 ± 0.7	0.008
PLT (125.0–350.0 × 10^9^ /L)	110.0 (62.0–186.0)	115.5 (65.0–186.8)	0.892
Inflammatory index			
PCT (0.02–0.05 ng/ml)^a^	0.29 (0.17–0.53)	0.20 (0.09–0.49)	0.082
CRP (mg/L)^b^	14.5 (6.1–33.4)	8.4 (3.5–17.7)	0.010

*ALB* albumin, *ALT* alaninetransaminase, *AST* aspartate aminotransferase, *TBIL* total bilirubin, *DBIL* direct bilirubin, *IBIL* indirect bilirubin, *INR*: international normalized ratio, *PTA* prothrombin activity, *WBC* white blood cell count, *N* neutrophils, *N% *neutrophil percentage, *PLT* platelet count, *PCT* procalcitonin, *CRP* C-reactive protein

^a^< 0.5 indicates low-risk severe systemic infection, 0.5—2.0 indicates moderate-risk severe systemic infection, ≥ 2.0 indicates high-risk severe systemic infection

^b^1 indicates low risk of cardiovascular disease, 1—3 indicates medium risk of cardiovascular disease, > 3 indicates high risk of cardiovascular disease, > 10 indicates the possibility of infection or inflammation

### Antimicrobial susceptibility testing results

The results of antimicrobial susceptibility testing of CRE strains are shown in Additional file 1: Table S2. All CRE isolates showed high resistance to cephalosporins, aztreonam, semisynthetic penicillin and levofloxacin (90%–100%). The rates of susceptibility to ciprofloxacin, trimethoprim-sulfamethoxazole, fosfomycin, gentamicin, and doxycycline were 9.3%, 16.28%, 16.28%, 27.91% and 27.91%, respectively. The CRE isolates showed the same susceptibility to ceftazidime/avibactam and amikacin (58.14%). They showed high susceptibility to polymyxin B (86.05%) and tigecycline (90.70%). Compared with carbapenem resistant *Escherichia coli* (CR-ECO) strains, carbapenem resistant *Klebsiella pnuemoniae* (CR-KPN) strains were more susceptible to polymixin B (100.00% *versus* 66.67%) and ceftazidime/avibactam (90.00% *versus* 25.00%), but more resistant to amikacin (60.00% *versus* 25.00%) and fosfomycin (90.00% *versus* 41.67%).

### Resistance genes

Distribution of resistance genes of 43 CRE strains were showed in Additional file 1: Table S3. Carbapenemase gene was positive in 83.72% (36/43) of the CRE isolations, including *bla*_*KPC-2*_ in 44.19% (19/43), *bla*_*NDM*_ in 13.95% (6/43) and multiple genes in 23.26% (10/43). Only 2 strains of CR-ECO were detected polymyxin resistance gene (*mcr-1*). Distribution of resistance genes of 36 carbapenemase-producing CRE (CP-CRE) strains were showed in Additional file 1: Table S4. *bla*_*KPC-2*_ was the most prevalent carbapenemase among CP-CRE (52.78%, 19/36), especially in CP-KPN (75.00%, 15/20). Compared with CP-KPN, *bla*_*NDM*_ was more common in CP-ECO (18.18%, 2/11) and other CP-CRE (60.00%, 3/5).

### Distribution of STs and carbapenemase

A total of 10 distinct STs were identified among 20 CR-KPN samples. As depicted in Table 4, ST11 was the dominant type (50%), followed by ST231 (10%). ST15, ST35, ST273, ST431, ST485, ST792, ST1933 and ST2472 took up to 5%, respectively. Only *bla*_*KPC-2*_ was detected in 10 ST11 strains. For 10 non-ST11 strains, *bla*_*KPC-2*_ was detected in 5 strains, *bla*_*KPC-2*_ + *bla*_*OXA-48*_ was detected in 2 strains, *bla*_*KPC-2*_ + *bla*_*NDM-5*_ was detected in 1 strain, *bla*_*KPC-2*_ + *bla*_*IMP-4*_ was detected in 1 strain, *bla*_*NDM-1*_ was detected in 1 strain.

**Table 4:** Distribution of STs and carbapenemase in CR-KPN

STs	N	Carbapenemase genes
ST11	10	*bla*_*KPC-2*_
Non-ST11	10	
ST15	1	*bla*_*KPC-2*_
ST35	1	*bla*_*KPC-2*_
ST231	2	*bla*_*KPC-2*_ + *bla*_*OXA-48*_
ST273	1	*bla*_*KPC-2*_ + *bla*_*NDM-5*_
ST431	1	*bla*_*KPC-2*_
ST485	1	*bla*_*KPC-2*_ + *bla*_*IMP-4*_
ST792	1	*bla*_*KPC-2*_
ST1933	1	*bla*_*KPC-2*_
ST2472	1	*bla*_*NDM-1*_

12 CR-ECO strains could be sorted into 7 STs with a scattered distribution (Table 5). ST648 complex was the most common type (33.33%), followed by ST10 complex (25%), ST95, ST410, ST2973, ST4985, ST6730 took up to 8.33%, respectively. Only *bla*_*KPC-2*_ was detected in 4 ST648 complex strains. For 3 ST10 complex strains, *bla*_*NDM*_*, bla*_*KPC-2*_ + *bla*_*IMP-4*_ and *bla*_*KPC-2*_ + *bla*_*NDM-9*_ + *bla*_*IMP-4*_ was detected in 1 strain, respectively. *bla*_*NDM*_ was detected in 1 ST2973 strain, *bla*_*KPC-2*_ + *bla*_*NDM*_ was detected in ST95, ST410 and ST6730 strain, respectively. No resistance gene was detected in ST4985 in the present study.

**Table 5:** Distribution of STs and carbapenemase in CR-ECO

STs	N	Carbapenemase genes
ST10 complex	1	*bla*_*NDM-1*_
	1	*bla*_*KPC-2*_ + *bla*_*IMP-4*_
	1	*bla*_*KPC-2*_ + *bla*_*NDM-9*_ + *bla*_*IMP-4*_
ST95	1	*bla*_*KPC-2*_ + *bla*_*NDM-5*_
ST410	1	*bla*_*KPC-2*_ + *bla*_*NDM-5*_
ST648 complex	4	*bla*_*KPC-2*_
ST2973	1	*bla*_*NDM-5*_
ST4985	1	*bla*_*KPC-2*_
ST6730	1	*bla*_*KPC-2*_ + *bla*_*NDM-1*_

## Discussion

Previous studies have shown that CRE colonization rates varied in different countries, regions, populations, and even different sampling sites [16–22]. In our study, the fecal carrying rate of CRE in patients with liver disease was lower than that of patients in the intensive care unit (ICU) and hematopoietic stem cell transplantation (HSCT) ward [23] and closer to that of outpatients [24, 25]. However, the carriage rate was 15.60% (17/109) in patients with ACLF in our study, which was similar with the rate in ICU and HSCT ward [23]. The rate of ACLF was 43.59% (17/39) and 18.11% (92/508) in the colonization group and non-colonization group respectively (as shown in Table 1). Among 39 patients with CRE colonization, 26 patients were detected at admission, 13 patients were detected during hospitalization. Since CRE colonization plays an important role in transmission of the pathogen through horizontal gene transfer in hospital, our findings support regular CRE screening for hospitalized patients with liver disease from the point of admission. The results of the present study showed that KPN and ECO were the main colonizing bacteria, a finding that is consistent with the prevalence of CRE strains from clinical specimens and the results of most colonization studies in China [10, 25, 26].

Risk factors for CRE colonization are numerous. Previous studies reported that severe underlying diseases, previous ICU hospitalization, long hospital stay, more invasive procedures, recent surgical history, previous infection history, long-term antibiotic use, long-term glucocorticoid use, and undergoing a transplant were risk factors for CRE colonization [16, 27–30]. However, in our study, no statistical significance in terms of the history of ICU hospitalization, invasive procedures, and steroid use between colonization and the non-colonization groups was found. This finding might have occurred because most of the previous research population focused on patients in ICU or transplant wards, but most of the population in our study were patients with simple past diagnosis and treatment experience. The present study revealed that ACLF, the history of pulmonary infection within past 3 months and the use of third generation cephalosporin/β-lactamases inhibitor within past 3 months were independent risk factors of CRE colonization in patients with liver disease. Among the 13 patients detected CRE colonization during hospitalization, 8 patients with ACLF, 6 patients combined with spontaneous peritonitis, 5 patients had pulmonary infection in prior 3 months, and all patients used antimicrobials, including third generation cephalosporin/β-lactamases inhibitor (Additional file 1: Table S1). In addition, it was found that when compared with the non-colonization group, the patients in the colonization group had poorer liver function, poorer blood coagulation function, and higher blood counts and high-sensitivity C-reactive protein. This finding suggested the severity of impaired liver function was associated with CRE colonization, which was consistent with the observation in liver transplant recipients [11, 31–35]. ESLD is associated with defects in the immune system, which increase the risk and severity of infections [36]. Pulmonary infection was one of the most common infections in patients with ESLD, and third generation cephalosporin/β-lactamases inhibitor was the recommended empirical antimicrobials [5]. Use of antimicrobials to treat infection might also disrupt the balance of intestinal flora and aggravate CRE colonization.

Previous studies showed that most CRE strains exhibit resistance to carbapenems because of carbapenemase production, among which KPC was predominant in KPN, and NDM was predominant in ECO, while OXA-48 was commonly found in Europe [37–40]. Consistent with the results from previous studies [40, 41], carbapenemase genes were detected in most CRE isolates (83.72%) in the present surveillance. The carrying rates of *bla*_*KPC-2*_ and *bla*_*NDM*_ were 83.33% and 33.33%, respectively. It was noteworthy that multiple carbapenemase genes were detected in 25.58% (11/43) strains, which was also reported in previous studies [10, 40, 42–44]. *bla*_*IMP-4*_ and *bla*_*OXA-48*_ were also detected in some CRE isolates as one of multiple carbapenemase genes. However, the carbapenemase gene involved in our study was not detected in 16.28% of CRE strains, indicating other carbapenem resistance mechanisms (such as hyperproduction of cephalosporinase, structural changes of binding sites, and others.) were present [10, 25].

The CRE strains in our study showed high resistance to carbapenems, β-lactams, fluoroquinolones, and aminoglycosides, while maintaining high sensitivity to polymyxin B and tigecycline, a finding that is consistent with the resistance data of CRE in China [45, 46]. It was reported that most carbapenem resistance organisms were also resistant to fluoroquinolones or aminoglycosides [47], which due to the integration of drug resistance genes [48, 49]. The resistance rates of CRE to aztreonam, cotrimoxazole and fosfomycin in our study were similar to the previous study in China [50], in which it reported that the resistance genes of multiple drugs could coexist and promote the prevalence of multidrug-resistant bacteria through horizontal transfer. Polymyxin B was currently used as a key antibiotic for treatment of carbapenem resistance organism infection [37, 51]. Consistent with the previous surveillance in China [52], the overall resistance rate of CREs to polymyxin B in our study was 13.95% (6/43 with 4 strains of CR-ECO and 2 strains of other CREs). The polymyxin resistance gene, *mcr-1*, was detected in only 2 ECO strains, indicating the existence of other resistance genes (*mcr-2*, *mcr-9*, *mgrB*, *phoP/phoQ* or *pmrA/pmrB*, among others.) or drug resistance mechanisms (capsular polysaccharide change, and others.) [53–55]. Tigecycline was another important treatment option for complicated intra-abdominal infection, skin and soft tissue infection and community acquired pneumonia caused by CRE [37, 56]. In agreement with the previous findings [10, 23, 25, 40], the overall resistance rate of CRE to tigecycline in our study was 2.33% (1/43, which included 1 strain of ECO). Ceftazidime/avibactam, a newer β-lactam agents that is active against CRE, had obvious effects on KPC-2 and OXA-48 producing CRE, but had poor activity on metalloenzymes-producing CRE [57–59]. Consistent with the previous reports, the resistance rate of KPN to ceftazidime/avibactam in our study was only 10% (2/20, which included one KPN carring *bla*_*NDM-5*_ and the other carring *bla*_*IMP-4*_), while the resistance rates of ECO and other CREs to ceftazidime/avibactam were 75% (9/12) and 63.64% (7/11), respectively. Metalloenzymes genes involved in our study were not detected in 6 CRE strains.

MLST was widely used in CRE, especially in KPN, and the typing results could indicate the genetic relationship between strains to a certain extent. As for KPN, ST11, ST15, ST258, and ST512 were the main popular categories in Europe and America [23, 60–62], while ST11 (mainly carrying *bla*_*KPC-2*_) was the main popular category of KPN in China [23, 25, 63, 64]. Similar to the above findings, 50% of KPNs in our study belonged to ST11 and all carried *bla*_*KPC-2*._ Unlike KPN, the ST types of ECO were scattered globally and varied greatly in different geographical areas. It showed that the prevalent CR-ECO were ST174 and ST648 that carried *bla*_*NDM-5*_ in India [65].However, in Korea, it showed that the prevalent CR-ECO were ST101 that carried *bla*_*NDM-1*_ [66]. Studies from some countries reported an association between ST10, ST410 and *bla*_*NDM*_ [67–69]. In our study, a total of 7 ST types were detected in 12 ECO strains, and the distribution was scattered. Most of ST10 carried *bla*_*NDM*_ and *bla*_*KPC-2*_, and all ST648 carried *bla*_*KPC-2*_. ESBL genes were not involved in our study. The above results were not completely consistent with other reports in China [41], which may be due to different geographical areas and the small number of our ECO isolates. Long-term and large-scale monitoring are required to clarify the epidemiology characteristics.

The present study was the first report focused on the prevalence, risk factors and molecular epidemiology of CRE colonization in patients with liver disease yet without liver transplantation as far as our knowledge. Moreover, both epidemiologic and laboratory data of patients were analyzed in our study. However, there are some limitations should also be acknowledged. First, this study was a single-center study with relatively small samples. The prevalence of CRE colonization and risk factors may not be generalizable to other hospitals or departments. Second, we did not observe whether the patients with CRE colonization developed into CRE infection. Third, this study only detected common carbapenemase genes but lacked further research on other resistance mechanisms.

## Conclusions

In conclusion, the present study revealed that CRE fecal carriage rates were much higher in patients with ACLF than in patients without liver failure. ACLF, the history of pulmonary infection within past 3 months and the use of third generation cephalosporin/β-lactamases inhibitor within past 3 months were independent risk factors of CRE colonization in patients with liver disease. Regular CRE screening for hospitalized patients with liver disease should be conducted to limit the spread of CRE strain. In addition, multi-center studies should be conducted to assess the prevalence, risk factors in patients with liver disease to support the enhanced infection control measures in this population.

## Supplementary Information

**Additional file 1.** Additional tables.

## Data Availability

The datasets used and/or analysed during the current study are available from the corresponding author on reasonable request.

## Abbreviations

CRE: Carbapenem Resistant *Enterobacteriaceae*
KPN: *Klebsiella Pnuemoniae*
ECO: *Escherichia Coli*
CR-KPN: Carbapenem Resistant *Klebsiella Pnuemoniae*
CR-ECO: Carbapenem Resistant *Escherichia Coli*
MALDI-TOF MS: Matrix-Assisted Laser Desorption/Ionization Time-of-Flight Mass Spectrometry
PCR: Polymerase chain reaction
MLST: Multilocus sequence typing
ACLF: Acute-on-chronic liver failure
MDR: Multidrug-resistant
ESLD: End-stage liver disease
ESBLs: Extended-spectrum β-lactamases
ICU: Intensive care units
HSCT: Hematopoietic stem cell transplantation
CRP: C-reactive protein
PCT: Procalcitonin
EUCAST: European Committee for Antimicrobial Susceptibility Testing
CLSI: Clinical and Laboratory Standards Institute
CP-CRE: Carbapenemase-Producing CRE
CP-KPN: Carbapenemase-Producing *Klebsiella Pnuemoniae*
CP-ECO: Carbapenemase-Producing *Escherichia Coli*
