# Supplementary material for: Risk factors and molecular epidemiology of fecal carriage of carbapenem resistant Enterobacteriaceae in patients with liver disease
Source: Ann Clin Microbiol Antimicrob. 2023 Jan 29;22:10. doi: 10.1186/s12941-023-00560-8 (PMC9884424; doi:10.1186/s12941-023-00560-8)
Supplement: Supplementary file 1 — Additional file 1. Additional tables. [file 12941_2023_560_MOESM1_ESM.docx]

**Additional file 1**

**Table 1** Details of 13 CRE colonization patients detected during hospitalization

| **No.** | **ACLF** | **Complication** | **Infection in prior 3 months** | **Invasive procedure during hospitalization** | **Antimicrobials using during hospitalization** |
| --- | --- | --- | --- | --- | --- |
| 3 | Y |  |  | Deep venous catheterization, Artificial liver support | Semi-synthetic penicillins/β-lactamase inhibitor |
| 5 | Y |  |  |  | Third generation cephalosporins/β-lactamase inhibitor |
| 6 | Y |  | Pulmonary infection | Deep venous catheterization, Artificial liver support | Third generation cephalosporins/β-lactamase inhibitor, Carbapenem |
| 7 | Y | Spontaneous peritonitis |  |  | Carbapenem, Fluoroquinolone |
| 9 | Y | Spontaneous peritonitis, Hepatorenal syndrome | Pulmonary infection |  | Third generation cephalosporins/β-lactamase inhibitor, Fluoroquinolone |
| 10 | Y | Spontaneous peritonitis, Hepatic encephalopathy | Pulmonary infection |  | Third generation cephalosporins/β-lactamase inhibitor, Carbapenem, Fluoroquinolone, Anti-fungal regimen |
| 13 | Y |  |  | Artificial liver support | Third generation cephalosporins/β-lactamase inhibitor, Fluoroquinolone |
| 16 | Y | Spontaneous peritonitis |  | Colonoscopy, Artificial liver support | Third generation cephalosporins/β-lactamase inhibitor, Carbapenem |
| 25 | N |  | Pulmonary infection, Urine tract infection | Liver aspiration/Biopsy | Semi-synthetic penicillins/β-lactamase inhibitor, Teicoplanin |
| 28 | N | Spontaneous peritonitis | Pulmonary infection | Paracentesis/Catheterization | Fluoroquinolone |
| 30 | N | Spontaneous peritonitis |  | Paracentesis/Catheterization | Semi-synthetic penicillins/β-lactamase inhibitor, Third generation cephalosporins/β-lactamase inhibitor, Tigecycline, Teicoplanin, Anti-fungal regimen |
| 31 | N |  |  | Artificial liver support | Semi-synthetic penicillins/β-lactamase inhibitor, Third generation cephalosporins/β-lactamase inhibitor, |
| 37 | N |  |  |  | Third generation cephalosporins |

**Table 2** Results of antimicrobial susceptibility of CRE strains

| **Antibiotic** | **All CRE (N = 43)** | | | **KPN (N = 20)** | | | **ECO (N = 12)** | | | **Other CRE (N = 11)** | |
| --- | --- | --- | --- | --- | --- | --- | --- | --- | --- | --- | --- |
|  | **R%** | **S%** | **R%** | | **S%** | **R%** | | **S%** | **R%** | | **S%** |
| CTX | 100.00 | 0.00 | 100.00 | | 0.00 | 100.00 | | 0.00 | 100.00 | | 0.00 |
| CAZ | 100.00 | 0.00 | 100.00 | | 0.00 | 100.00 | | 0.00 | 100.00 | | 0.00 |
| FEP | 100.00 | 0.00 | 100.00 | | 0.00 | 100.00 | | 0.00 | 100.00 | | 0.00 |
| CPS | 100.00 | 0.00 | 100.00 | | 0.00 | 100.00 | | 0.00 | 100.00 | | 0.00 |
| CXM | 100.00 | 0.00 | 100.00 | | 0.00 | 100.00 | | 0.00 | 100.00 | | 0.00 |
| CFZ | 100.00 | 0.00 | 100.00 | | 0.00 | 100.00 | | 0.00 | 100.00 | | 0.00 |
| PIP | 97.67 | 0.00 | 100.00 | | 0.00 | 91.67 | | 0.00 | 100.00 | | 0.00 |
| P/T | 97.67 | 0.00 | 100.00 | | 0.00 | 91.67 | | 0.00 | 100.00 | | 0.00 |
| AMC | 95.35 | 2.33 | 100.00 | | 0.00 | 83.33 | | 8.33 | 100.00 | | 0.00 |
| IMP | 79.07 | 11.63 | 80.00 | | 10.00 | 66.67 | | 25.00 | 90.91 | | 0.00 |
| MRP | 88.37 | 9.30 | 95.00 | | 5.00 | 75.00 | | 25.00 | 90.91 | | 0.00 |
| ETP | 100.00 | 0.00 | 100.00 | | 0.00 | 100.00 | | 0.00 | 100.00 | | 0.00 |
| LEV | 90.70 | 6.98 | 95.00 | | 0.00 | 91.67 | | 8.33 | 81.82 | | 18.18 |
| TGC | 2.33 | 90.70 | 0.00 | | 90.00 | 8.33 | | 91.67 | 0.00 | | 90.91 |
| PB | 13.95 | 86.05 | 0.00 | | 100.00 | 33.33 | | 66.67 | 18.18 | | 81.82 |
| AMK | 41.86 | 58.14 | 60.00 | | 40.00 | 25.00 | | 75.00 | 27.27 | | 72.73 |
| DOX | 55.81 | 27.91 | 40.00 | | 30.00 | 66.67 | | 33.33 | 72.73 | | 18.18 |
| SXT | 83.72 | 16.28 | 90.00 | | 10.00 | 66.67 | | 33.33 | 90.91 | | 9.09 |
| FO | 74.42 | 16.28 | 90.00 | | 5.00 | 41.67 | | 33.33 | 81.82 | | 18.18 |
| CIP | 88.37 | 9.30 | 95.00 | | 0.00 | 91.67 | | 8.33 | 72.73 | | 27.27 |
| GEN | 72.09 | 27.91 | 80.00 | | 20.00 | 66.67 | | 33.33 | 63.64 | | 36.36 |
| ATM | 93.02 | 4.65 | 95.00 | | 0.00 | 100.00 | | 0.00 | 81.82 | | 18.18 |
| CZA | 41.86 | 58.14 | 10.00 | | 90.00 | 75.00 | | 25.00 | 63.64 | | 36.36 |

Abbreviations: CTX: cefotaxime; CAZ: ceftazidime; FEP: cefepime; CPS: cefoperazone/sulbactam; CXM: cefuroxime; CFZ: cefazolin; PIP: piperacillin; P/T: piperacillin/tazobactam; AMC: amoxicillin-clavulanate; IMP: imipenem; MRP: meropenem; ETP: ertapenem; LEV: levofloxacin; TGC: tigecycline; PB: polymixin B; AMK: amikacin; DOX: doxycycline; SXT: trimethoprim-sulfamethoxazole; FO: fosfomycin; CIP: ciprofloxacin; GEN: gentamicin; ATM: aztreonam; CZA: ceftazidime/avibactam.

**Table 3** Distribution of different carbapenemase genes in 43 CRE strains

| **Species** | **Strains screened,**  **n (%)** | **bla_KPC-2_,**  **n (%)** | **bla_NDM_,**  **n (%)** | **Multiple genes,**  **n (%)** | **Any genes,**  **n (%)** |
| --- | --- | --- | --- | --- | --- |
| KPN | 20(46.51) | 15(34.88) | bla_NDM-1_,  1(2.33) | bla_KPC-2_+bla_NDM-5_,  1(2.33) | 20(100.00) |
|  |  |  |  | bla_KPC-2_+bla_OXA-48_,  2(4.65) |  |
|  |  |  |  | bla_KPC-2_+bla_IMP-4_,  1(2.33) |  |
| ECO | 12(27.91) | 4(9.30) | bla_NDM-1_,  1(2.33) | bla_KPC-2_+bla_NDM-1_,  1(2.33) | 11(91.67) |
|  |  |  | bla_NDM-5_,  1(2.33) | bla_KPC-2_+bla_NDM-5_,  2(4.65) |  |
|  |  |  |  | bla_KPC-2_+bla_IMP-4_,  1(2.33) |  |
|  |  |  |  | bla_KPC-2_+bla_NDM-9_+bla_IMP-4_,  1(2.33) |  |
| Other CRE | 11(25.58) |  | bla_NDM-1_,  3(6.98) | bla_KPC-2_+bla_NDM-1_,  1(2.33) | 5(45.45) |
|  |  |  |  | bla_KPC-2_+bla_IMP-4_,  1(2.33) |  |
| Total | 43(100.00) | 19(44.19) | 6(13.95) | 11(25.58) | 36(83.72) |

**Table 4**  Distribution of different carbapenemase in 36 CP-CRE strains

| **Carbapenemase** | **CP- CRE,**  **n (%)** | **CP-KPN,**  **n (%)** | **CP- ECO,**  **n (%)** | **Other CP-CRE,**  **n (%)** |
| --- | --- | --- | --- | --- |
| KPC-2 | 19(52.78) | 15(75.00) | 4(36.36) | 0(0.00) |
| NDM^a^ | 6(16.67) | 1(5.00) | 2(18.18) | 3(60.00) |
| KPC-2+NDM^a^ | 5(13.89) | 1(5.00) | 3(27.27) | 1(20.00) |
| KPC-2+OXA-48 | 2(5.56) | 2(10.00) | 0(0.00) | 0(0.00) |
| KPC-2+IMP-4 | 3(8.33) | 1(5.00) | 1(9.09) | 1(20.00) |
| KPC-2+NDM^a^+IMP-4 | 1(2.78) | 0(0.00) | 1(9.09) | 0(0.00) |
| Total | 36(100.00) | 20(55.56) | 11(30.56) | 5(13.89) |

a : NDM-1,NDM-5.
